# Highly efficient and rapid dechlorination of polyvinyl chloride via microwave pyrolysis

**DOI:** 10.1098/rsta.2024.0064

**Published:** 2025-05-22

**Authors:** Chai Siah Lee, Mohamed Adam, John P. Robinson, Eleanor R. Binner

**Affiliations:** ^1^ Faculty of Engineering, University of Nottingham, Nottingham, UK; ^2^ Halocycle Ltd., Hull, UK

**Keywords:** polyvinyl chloride, dechlorination, microwave pyrolysis, plastic recycling

## Abstract

Polyvinyl chloride (PVC) waste recycling is challenging due to its high chlorine content, which generates hazardous chlorinated pollutants if treated improperly. A safe and promising PVC dechlorination method is urgently needed to address this issue. Several dechlorination methods have been reported at the laboratory scale; however, each method has its downsides, and none has been proven at the commercial scale. We present, for the first time in the literature, an effective microwave pyrolysis process that can dechlorinate PVC rapidly without the requirement of a solvent/microwave absorber. High dechlorination efficiency up to 99.6% was achieved within 96 s. This process releases hydrogen chloride and generates hydrocarbon-containing liquid and a dechlorinated residue. Dielectric analysis revealed that the untreated PVC was readily heated under microwaves due to the polar chlorine group in its structure. Thermogravimetric analysis confirmed that there were two pyrolysis stages and dechlorination was achieved after the first pyrolysis stage. Fourier-transform infrared (IR) analysis showed that all the bands corresponding to the stretching of C-Cl bonds were not detected in the dechlorinated residue. All these results prove that microwave pyrolysis is a promising process for PVC dechlorination, and it could be the game changer that makes PVC recycling commercially viable.

This article is part of the discussion meeting issue ‘Microwave science in sustainability’.

## 1. Introduction

Polyvinyl chloride (PVC) is the third most used polymer with a global production of 50.7 Mt, estimated in 2021 [1]. Its popularity is attributed to its versatility, durability, low thermal conductivity and low production costs [2]. PVC can be found in a rigid or flexible form, depending on the amount of plasticizers added [3]. Rigid PVC contains little to no plasticizer in it; thus, it is hard and difficult to bend. On the other hand, flexible PVC usually contains phthalate plasticizers, making it soft and easy to bend. PVC plastics are widely used in many applications, such as building and construction, pipes, floor coverings, medical devices, cables and packaging [4].

Due to the large amount of PVC waste generated, there is increasing attention to recycling PVC globally [5]. However, PVC recycling remains very challenging because of its high chlorine content (approximately 56 wt% of the polymer weight for rigid PVC [6]), which generates chlorinated pollutants, and high levels of hazardous phthalate plasticizers (up to 40 wt% of the polymer weight [7]) in flexible PVC. It has been reported that landfill, incineration and mechanical recycling are the common PVC waste management practices [8–12]. However, landfills and incineration have fallen into economic and environmental difficulties due to land shortages and potential hazards caused by the formation of chlorine-containing compounds (e.g. polychlorinated dibenzodioxins, polychlorinated dibenzofurans and polychlorinated biphenyls [13]) at high temperatures during incineration and the leaching of harmful plasticizers that might migrate into the soil which poses different environmental and health risks [14]. For mechanical recycling, the heat and pressure cycles involved in the crushing and melting processes typically degrade the polymer’s quality, which results in lower-value materials and limits the number of PVC reprocessing cycles [6,9]. As a result, PVC is known as the most challenging to recycle and environmentally damaging plastic.

Therefore, a safe and environmentally friendly approach to treat and recycle PVC waste is urgently needed to address the negative environmental and health effects caused by PVC waste. Chemical recycling has received more attention and emerged as a promising alternative to other plastic waste management practices because it is suited to decompose or depolymerize all types of plastic waste [15]. In addition, it can break down the polymer into oligomers/monomers or naphtha-like chemicals that can be used to make a new polymer, secondary materials or feedstocks to make chemicals and fuels [16]. For the PVC chemical recycling process, the polymer cannot be converted into its oligomers or monomers, but it is dechlorinated and broken down into valuable small molecular chemicals, fuels and feedstock [15]. In any potential PVC chemical recycling process, dechlorination is the essential primary step. Several PVC chemical recycling dechlorination methods reported in the literature and their advantages and drawbacks are listed in table 1.

**Table 1.** A review of the PVC dechlorination methods reported in the literature comparing their advantages and drawbacks.

PVC dechlorination method	advantage(s)	drawback(s)
non-thermal plasma ([1] for rigid PVC)	a dechlorination efficiency of 98.3% could be achieved at 180 W of power at 280°C.	a treatment time of up to 40 minutes is required.
dehalogenation using organic solvent mixture ([17] for rigid and flexible PVC)	dehalogenation could be achieved at room temperature by converting PVC plastics into carbonaceous materials.	a treatment time of up to a few hours is required. using organic solvents such as dimethylformamide is of great environmental concern [18].
hydrothermal treatment ([2,19] for rigid PVC and [20–22] for flexible PVC)	dechlorination efficiencies of 90.2 to 98.2% could be achieved at temperatures between 250 and 300°C.	pressure up to a few MPa and one hour to a few hours of treatment time is required.
conventional pyrolysis ([13,23–26] for rigid PVC)	the process is simple and cost-effective. dechlorination efficiencies of 90 to 94.6% could be achieved under atmospheric pressure at temperatures between 260 and 400°C with treatment time between 9 and 30 min. the treatment time is shorter than non-thermal plasma, solvent dehalogenation and hydrothermal methods.	the dechlorination efficiency is lower than the non-thermal plasma method and similar to or lower than the hydrothermal treatment method.
microwave pyrolysis ([27] for a mixture of rigid and flexible PVC)	a dechlorination efficiency of 90% could be achieved under atmospheric pressure at 400°C after 35 min of treatment time.	a microwave absorber (e.g. activated carbon or Zn-Mn ferrite) is needed to achieve 90% dechlorination efficiency.

The literature review in table 1 has demonstrated that each reported PVC dechlorination method has downsides. For example, longer treatment time (more than half an hour) is needed for non-thermal plasma, solvent dehalogenation and hydrothermal methods; this potentially leads to the requirement of a larger equipment size, which increases the capital cost and facility footprint. Another drawback is the requirement of a harmful solvent for the solvent dehalogenation method, which may increase the process complexity and the process cost due to the requirement of a downstream process to separate the solvent if the products are contaminated with the solvent. Furthermore, another waste stream is created if the solvent cannot be recycled and reused in the process. Even though table 1 shows that conventional pyrolysis is more widely investigated, probably due to its simplicity and cost-effectiveness [28], its dechlorination efficiency requires improvement. Consequently, there is an urgent demand to improve the current methods or develop a new and promising PVC dechlorination method that has the advantages of (i) high dechlorination efficiency (preferably greater than 99%), (ii) short treatment time (preferably in a few minutes or less), and (iii) without the use of a hazardous solvent/additive.

Microwave heating has been reported as a promising technique for facilitating the breakdown of polymeric chains to achieve chemical recycling [29] because the heating is achieved rapidly through the interaction of the electromagnetic field with the material at molecular and/or sub-molecular (i.e. mobile electrons) levels [30]. Consequently, the whole material can be heated rapidly and uniformly; this potentially delivers the advantages in energy efficiency, processing time and product quality. As listed in table 1, the application of microwave pyrolysis to dechlorinate PVC was reported in 2006 but a microwave absorber was used in that process to achieve 90% dechlorination efficiency [27]; this potentially complicated the process and increased the processing cost if a downstream separation or purification process is required. Adam *et al*. [30] reported that a microwave absorber/susceptor is likely to be needed for effective microwave heating if microwave heating is applied to a polymer without polar segments such as polystyrene. PVC contains a polar chloride group within its structure indicating its capability to interact with the alternating microwave field without an absorber.

In this work we aimed to assess the potential for microwave pyrolysis to dechlorinate and decompose PVC without the addition of either a microwave absorber or a chemical solvent, and to investigate the products for their potential use as valuable products. The objectives were to evaluate the efficiency of microwave heating to heat and dechlorinate PVC across the dechlorination temperature range (175 to 350°C), understand the effect of treatment time on dechlorination efficiency and characterize the products to understand their potential to be used in future applications.

## 2. Material and methods

### (a) Materials

PVC cable sleeves with 4 mm diameter were sourced from RS Components Ltd. (UK) and used as the flexible PVC material depolymerized in this study. Flexible PVC was selected for this study because it accounts for 58 to 70% of post-consumer PVC waste [11] and fewer PVC dechlorination studies were conducted with flexible PVC. The PVC was cut into a 2 to 3 mm curved shape to achieve a denser packing of cut PVC in the reactor. For every experiment, 12.6 g of the cut PVC was packed into the reactor with a sample height between 3.7 and 4.0 cm for consistent packing density and homogeneous heating within the microwave heating zone.

### (b) Microwave pyrolysis process

**Figure 1.**
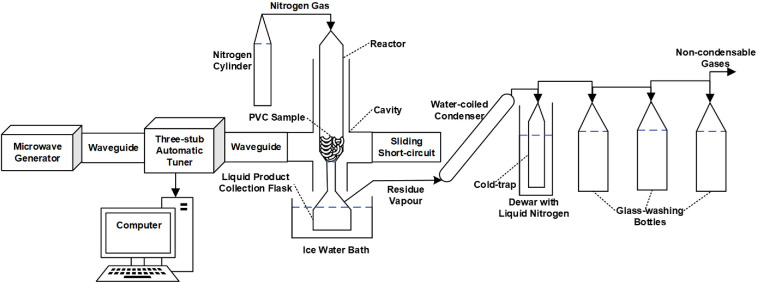
Microwave pyrolysis experimental apparatus for PVC dechlorination.

A schematic diagram of the experimental apparatus for microwave dechlorination of PVC is shown in figure 1.

The microwave power was provided by a 2.45 GHz 2 kW microwave generator and then transmitted to the single-mode cavity via WR340 waveguides. A schematic of the microwave cavity used in the heating experiments, with dimensions, is presented in electronic supplementary material, fig. S1. A sliding short-circuit and a three-stub motorized Homer automatic tuner was used for impedance matching to improve the microwave power delivery efficiency. The tuner also sampled and analysed the microwave power signal to determine the input and reflected power as well as the frequency. Before commencing the experiment, a vector network analyser was used to position the sliding short-circuit and tuning stubs for optimal settings that maximized the absorbed power. This procedure is commonly known as cold matching.

A quartz reactor tube with an opening at the bottom of the reactor was filled and packed with the cut PVC sample. A porous thin ceramic fibre (2.6 cm diameter) was used to hold the PVC sample at the reactor opening, preventing it from dropping into the collection flask. The reactor with the packed PVC sample and the products generated from the process are shown in electronic supplementary material, fig. S2. The reactor with the sample was inserted into the cavity through the top choke. Nitrogen carrier gas was supplied from the top of the reactor at 0.2 l min^–1^ regulated by a gas flow meter. The system was purged with nitrogen flow for at least 10 min before starting the run to create an oxygen-free environment. The microwave generator with a set input power (800 W) was turned on to start the experiment. The microwave input power and reflected power were recorded via HomSoft software (S-TEAM Homer windows visualization and control software for easy control and monitoring of Homer auto-tuner) throughout the experiment. The absorbed power was calculated by subtracting the reflected power from the input power, as measured by the automatic tuner. The specific energy was calculated as the microwave-absorbed energy (average absorbed power multiplied by treatment time) per mass of the sample treated. Consequently, the calculated specific energy did not account for any power potentially absorbed by the waveguide and cavity walls; however, as these components were constructed from aluminium, a metal with high electrical conductivity, their capacity to absorb microwave power was expected to be very limited.

Temperature measurement is inherently difficult during microwave processing. The insertion of a metallic thermocouple into the sample can affect the microwave field configuration and result in localized heating. Skin heating may also take place on the probe’s surface. Both effects will result in inaccurate temperature measurements. Therefore, a non-contact IR measurement device (Optris Xi80 thermal imaging camera) was selected to measure the external reactor wall temperature. It was located on the rear choke opening of the cavity; the device is capable of measuring and recording temperatures in the range −20 to 900°C, with a distance-to-spot-size ratio of up to 190:1.

The bottom of the reactor was connected to a collection flask immersed in an ice water bath (0°C) for the condensation of vapour into liquid and the main liquid product was collected in the flask. This was followed by a water-coiled condenser and then a cold trap immersed in a dewar containing liquid nitrogen for further condensation of residue vapour and a small amount of liquid product was collected in the cold trap. Afterwards, the residue vapour was passed through three glass-washing bottles that contained deionized water for the capture of hydrogen chloride (HCl) vapour.

After the experiment, the cold trap was removed from the dewar and left at room temperature for 15 min for the evaporation of trapped HCl in the cold trap, and the released HCl was captured by the glass washing bottles. Following this, the weights of the solid product left in the reactor tube (termed as dechlorinated (de-Cl) residue) and the collected liquid product were measured for the calculation of the solid and liquid yields. The water in the glass washing bottles was neutralized with sodium hydroxide to pH 7 for the calculation of the HCl yield. The chlorine (Cl) removal efficiency was calculated by the following steps: (i) obtaining the original chlorine concentration in the untreated PVC with X-ray fluorescence (XRF) analysis, (ii) converting the original chlorine concentration to original HCl concentration, and (iii) comparing the experimental HCl yield with the original HCl concentration in the untreated PVC.

### (c) Characterization of untreated PVC and the pyrolysis products

Here we describe the analytical work that was performed to characterize the untreated PVC, the de-Cl residue and the liquid product collected from the experiments that produced the highest chlorine removal efficiency.

#### (i) Differential scanning calorimetry (DSC)

The glass transition temperature (T_g_) of the untreated PVC was determined via DSC. The analysis was carried out using a Discovery DSC 2500 system manufactured by TA Instruments. The measurements were conducted within the range of −20 to 120°C at a heating rate of 10°C min^–1^ [31].

#### (ii) XRF analysis

The chlorine concentration in the untreated PVC and the chemical elements in the de-Cl residue were determined according to Zhang *et al*. [22] and Chen *et al*. [32] using a handheld Niton XL3T 950 GOLDD + XRF analyser mounted on a mobile phase stand. At least three measurements were taken for the same sample, and the sample cup was moved to a different spot for each measurement.

#### (iii) Dielectric properties analysis

Dielectric property measurements of the untreated PVC and de-Cl residue were carried out using a cavity perturbation technique following the method described in [33]. This technique was chosen because it is a well-established method to measure the dielectric properties of solids at microwave frequencies and can take high-temperature readings [34]. The system consists of a cylindrical copper cavity connected to a vector network analyser, which measures the frequency shift and change in quality factor relative to the empty resonating cavity when a sample is introduced. The sample was loaded into a quartz tube with a packing density of 0.54 to 0.6 g cm^–3^ and then held in a conventionally heated furnace above the cavity. The sample was heated gradually in the furnace with an average heating rate of 1.7°C min^–1^ until the temperature set point was reached, then held at the temperature set point for 60 s. The tube containing the sample was then moved into the cavity in less than 1 s with a high-speed motor, and the properties were determined in approximately 10 s at 2450 MHz, which is the frequency of the microwave heating equipment used in this study. The process was repeated over a temperature range of 20 to 400°C by holding the sample in the furnace at set temperature intervals of 20°C then moving it into the cavity for measurement.

#### (iv) Thermogravimetric analysis (TGA)

The weight-loss behaviours of the untreated PVC and de-Cl residue were obtained with a TGA Q500A (TA Instruments). Typically, 15 mg of sample were loaded on to the TGA pan and then heated from room temperature to 550°C at a heating rate of 10°C min^–1^ with 50 ml min^–1^ of nitrogen flow.

#### (v) Attenuated total reflectance Fourier-transform IR (ATR-FTIR) analysis

This analysis was used to identify the chemical bonds and structures of the untreated PVC and de-Cl residue. The ATR-FTIR spectra were recorded on a Bruker Alpha II FTIR spectrometer equipped with a monolithic diamond crystal. The wavenumber was set from 4000 to 400 cm^−1^, the resolution was set to 4 cm^−1^, and the number of scans was set to 32 for each measurement. The air background spectrum was collected every 30 minutes.

#### (vi) X-ray powder diffraction (XRD) analysis

The crystalline compound in the de-Cl residue was identified through XRD analysis. It was performed using a D8-Advance X-ray diffractometer (Bruker, Germany) using Cu Kα radiation (λ = 1.54 Å) in a 2θ range between 10° and 80°, and then the diffraction patterns were analysed by DIFFRAC.EVA software with the Powder Diffraction File database to obtain the composition of the material.

#### (vii) Elemental analysis

The carbon (C), hydrogen (H) and nitrogen (N) contents of the de-Cl residue were analysed using an elemental analyser (LECO, CHN628S).

#### (viii) Gas chromatography-mass spectrometry (GC-MS)

The identification of the main chemical compounds in the liquid product was conducted via GC-MS by Intertek Wilton Chemicals and Materials Testing Labo 118 mg of the liquid product was dissolved in 2 ml of chloroform before being analysed by GC-MS. The GC-MS system was Agilent 8890 GC with Agilent 5977B Series Mass Selective Detector. The sample was injected into the GC-MS at 350°C and the column (30 m × 0.25 mm × 0.25 µm) used was ZB−5HT. The helium carrier gas was supplied at a split ratio of 20:1 with a flow rate of 1 ml min^–1^. The oven programme was as follows: (i) hold for 5 min at 30°C, (ii) ramp to 350°C at 10°C min^–1^, and (iii) hold for 15 min at 350°C. The chemical compounds were detected by the MS instrument in electron ionization mode at 70 eV, then the compounds were identified using the MS Workstation with the National Institute of Standards and Technology mass spectral library.

## 3. Results and discussion

### (a) Microwave dechlorination of PVC

**Figure 2.**
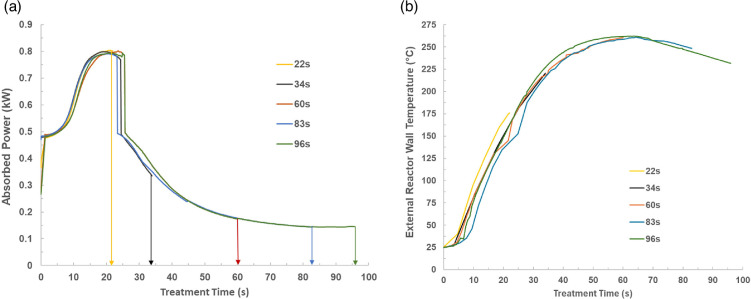
The (a) absorbed power profiles and the (b) external reactor wall temperature profiles achieved during microwave dechlorination of PVC at different treatment times.

The XRF analysis revealed that the chlorine concentration in the untreated PVC was 34 ±0.34% of the polymer weight. A microwave pyrolysis process was applied to decompose and dechlorinate PVC with constant microwave input power at 800 W with a variation of treatment time from 22 to 96 s. The microwave-absorbed power profiles and the external reactor wall temperature profiles at different treatment times are presented in figure 2.

As shown in figure 2*a*, the absorbed power increased rapidly with increasing treatment time, reached the peak at 20 s (defined as the fast heating stage), then dropped as treatment time progressed (slow heating stage) and finally reached a plateau (stabilization stage) after 85 s. The fast heating stage reveals that PVC absorbs microwave power readily without the requirement of a microwave absorber; this is probably due to the presence of the polar chlorine group and the polar phthalate plasticizer within the PVC structure (more details are provided in §3b(ii)). The slow heating stage indicates that dechlorination has started and some chlorine and plasticizer have been removed from the polymer. The stabilization stage is reached when most or all of the chlorine and plasticizer have been removed from the PVC, leaving a de-Cl residue that does not heat any further. This indicates the completion of the first pyrolysis stage (more details in §3b(iii)) and correlates with the identification of cyclic and aromatic hydrocarbons in the liquid product, which are the pyrolytic products formed during and after the first pyrolysis stage (more details in §3c).

The absorbed power profiles correspond to the three stages observed on the temperature profiles in figure 2*b*. Initially, the temperature increased rapidly with treatment time until approximately 50 s (first stage) and then was constant between 50 and 70 s around 260°C (second stage); this corresponds to the aforementioned fast and slow heating stages. After 70 s, it is expected that most of the chlorine and plasticizer has been removed from the PVC; thus, the temperature started to drop accordingly (third stage) corresponding to the stabilization stage on the absorbed power profiles.

The temperature peak was reached at 50 s, which occurred later than the absorbed power peak at 20 s. This can be attributed to the heat loss and the time taken for heat transfer from the heated sample to the external reactor wall surface. Even though the highest external reactor wall temperature measured during the experiment was 261°C, it is thought that the real temperature in the reactor centre was above 261°C and most probably higher than 300°C. This is because the PVC sample was selectively heated in a cold surrounding whilst the reactor tube was heated in the presence of a thermal gradient which is the driving force for outward heat transfer from the reactor centre to the colder surroundings.

**Figure 3.**
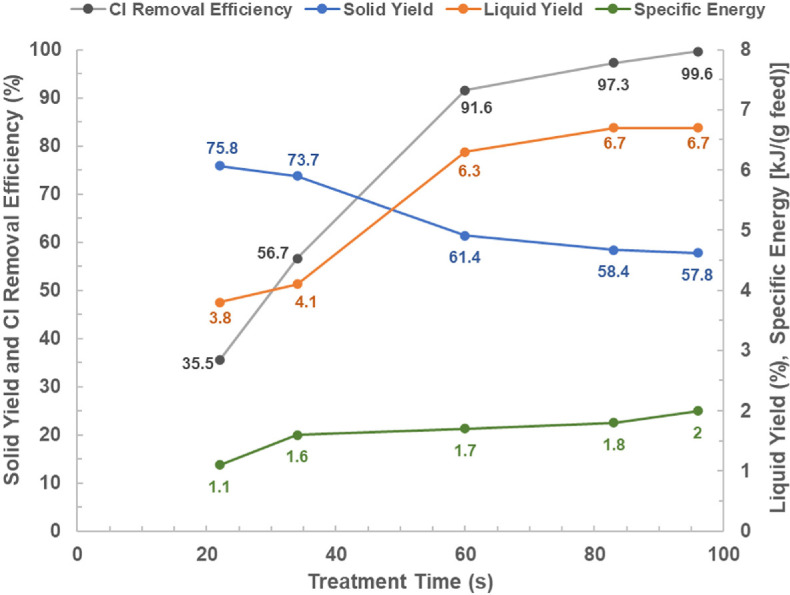
Microwave dechlorination of PVC with the effect of treatment time on Cl removal efficiency, solid yield, liquid yield and specific energy.

The results for the effects of treatment time on the solid yield, liquid yield, chlorine removal efficiency and specific energy are presented in figure 3.

Figure 3 clearly shows that the Cl removal efficiency and liquid yield increased with a longer treatment time; in addition, the solid yield decreased with the treatment time. The decrease in solid yield with the treatment time is because of PVC dechlorination, which removes chlorine from the polymer [13,35], and PVC decomposition which converts or transforms the polymer into a liquid product containing hydrocarbons and plasticizer (more details can be found in §3c).

At 83 s which is approaching the stabilization stage, most of the chlorine has been removed with 97.3% Cl removal efficiency, 6.7% liquid yield and 58.4% solid yield. The best results were attained at 96 s with the achievements of 99.6 ± 0.2% Cl removal efficiency, 6.7 ± 0.1% liquid yield, and 57.8 ± 0.9% solid yield at 2 ± 0.3 kJ g(feed)^–1^ specific energy. The experiment with a treatment time of 96 s was repeated three times with a standard deviation of less than 1%. As the product yields and Cl removal efficiency at 83 and 96 s were very close, 83 s was highlighted as a reasonable treatment time to remove most of the chlorine from this PVC.

### (b) Characterization and comparison between the untreated PVC and de-Cl residue

#### (i) Physical appearance

Electronic supplementary material, fig. S2 presents the untreated PVC and de-Cl residue, and it shows that the de-Cl residue was a black solid. This is consistent with another study whereby the PVC turned into black pyrolytic char when 89.9% Cl removal efficiency was achieved via a conventional pyrolysis process [13].

#### (ii) Dielectric properties via cavity perturbation technique

To understand the heating behaviour observed in §3a, the dielectric properties of the untreated PVC and de-Cl residue were measured. Dielectric properties define the interaction of a material with an applied electromagnetic field, thereby determining its heating efficiency. The dielectric constant (ε′) is a measure of the material’s ability to store electrical energy [30]. In addition, the loss factor (ε″) indicates how well a material converts electromagnetic energy into heat and is directly proportional to the power dissipation density [36]. The loss tangent (tan δ) is defined as the ratio of the loss factor to the dielectric constant and is commonly used to compare the efficiency of energy conversion among different materials [30].

**Figure 4.**
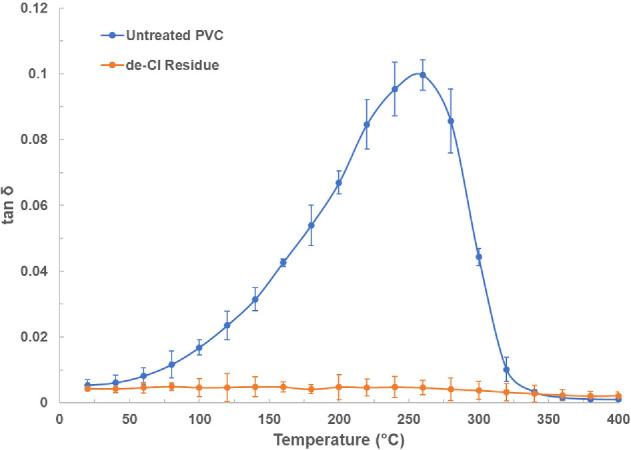
Loss tangent (tan δ) at 2450 MHz for the untreated PVC and de-Cl residue. Each dielectric measurement was conducted in triplicate, with the standard deviation calculated and represented as error bars.

The dielectric constant and loss factor results are shown in electronic supplementary material, fig. S3, while the loss tangent results are presented in figure 4. Due to the difference in sample particle size and shapes, there was difficulty in achieving the same packing density for dielectric measurements; therefore, the loss tangent results that consider both the dielectric constant and loss factor were used for the comparison of their dielectric heating efficiency.

The value of T_g_ is an important measure for understanding certain dielectric behaviours of untreated PVC in figure 4. For untreated flexible PVC, DSC measurements revealed a T_g_ value of 55 ± 0.5°C, which is lower than the literature-reported T_g_ (76 to 77°C) [31,37] values for rigid PVC. This is because the plasticizer in it has a lower molecular weight than PVC with a relatively large branched molecular structure; therefore, the free volume around the plasticizer is larger than PVC [38]. Consequently, the presence of plasticizer in PVC increases the free volume per total volume in the system due to a proportional increase in branch points, which is an increase of free space within the polymer; hence, the intermolecular forces between PVC chains are weaker lowering the T_g_ [39].

The flexible PVC investigated in this study contains PVC and phthalate plasticizer compounds, verified by the FTIR and GC results in §§3b(iv) and 3c, respectively. As the PVC compound is a long polymer with high molecular weight, its molecular rotation with the electromagnetic field is impeded by its complex structure. However, the chlorine group (−Cl) within the repeating units of the polymer structure is polar and, therefore, it can act as the primary contributor to molecular movement and interaction under the microwave field leading to microwave heating through the dipolar dispersion mechanism [40]. The presence of phthalate plasticizer in this PVC may contribute to microwave heating because it can help increase the ability of the polymer chains to interact with the electromagnetic field by reducing the intermolecular forces of the PVC chains [30]. Furthermore, as phthalates are polar compounds, the permanent or induced dipoles within the material will rotate to align themselves with the oscillating electromagnetic field which enables the material to be heated by microwaves through the dipolar loss mechanism [40].

Figure 4 shows that the loss tangent of untreated PVC increased slowly with temperature below 55°C, which was the T_g_ of this PVC. This is due to the extended and complex covalently bonded molecular structure, which restricts molecular rotation in response to the electromagnetic field when the temperature is below the T_g_ [30]. After exceeding T_g_, the loss tangent increased rapidly from 0.008 to 0.1 with increasing temperature and then peaked at 260°C; this corresponds to the rapid increase of absorbed power within 20 s in figure 2*a*. This is because when the temperature reaches the T_g_ of this PVC, the polymer changes from a brittle glassy state to an elastic rubbery state. Then, it is expected that the specific volume of the polymer will increase significantly due to this transition. This will expand the gap between the molecular chains allowing the polar molecules to have higher mobility to rotate and align themselves with the oscillating electromagnetic field [40]. Therefore, the dielectric behaviour of PVC observed between 55 and 260°C in figure 4 is due to the presence of the polar chlorine groups and possibly the polar phthalate plasticizer, which allows the PVC to be heated directly.

After the peak, the loss tangent dropped quickly between 260 and 320°C. This is probably due to one or a combination of the following three reasons: thermal decomposition of PVC with a change in chemical composition or reduction in sample mass; dissociation of the chlorine group from the polymer backbone due to dechlorination; removal of plasticizer from the PVC. After 320°C, the loss tangent was nearly zero, possibly indicating the completion of the dechlorination stage leaving a dechlorinated residue.

The loss tangent profile of the untreated flexible PVC in this study is in good agreement with the loss tangent profile of a flexible PVC reported in a recently published work [30]. For the de-Cl residue, the loss tangent was very low, with values close to zero within the temperature range from 20 to 400°C, which is probably because the polar chlorine group and plasticizer had been removed by microwave pyrolysis.

#### (iii) Weight-loss profiles (pyrolysis behaviour) via TGA

**Figure 5.**
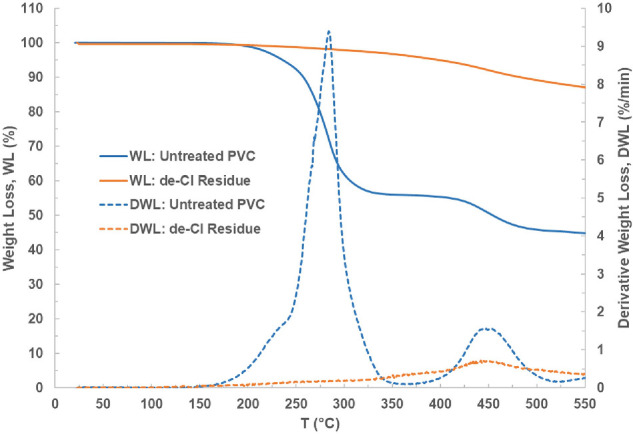
The weight-loss (WL) and derivative weight-loss (DWL) profiles from room temperature to 550°C for the untreated PVC and de-Cl residue.

The weight-loss and derivative weight-loss profiles for the untreated PVC and de-Cl residue, from room temperature to 550°C are presented in figure 5. The pyrolysis behaviour can be explained from these profiles. It can be seen that the untreated PVC showed two distinct pyrolysis stages with 44% as the first weight loss between 175 and 350°C (first pyrolysis stage) and 11.2% as the second weight loss between 410 and 525°C (second pyrolysis stage).

The literature reports that the main reaction in the first pyrolysis stage occurring between 175 and 350°C is polymer dechlorination forming de-Cl residue (solid) and conjugated polyene (liquid) with the release of volatiles that mainly consist of HCl [13,35]. Most of the chlorine can be completely removed after the first stage, while the second pyrolysis stage happening between 350 and 525°C is the cracking and decomposition of the de-Cl residue forming aromatic and condensed ring hydrocarbons [41,42].

The first weight loss (44%) for the PVC selected in this study is lower than the weight loss (65 to 68% [32,41]) reported for the rigid PVC in the literature because the selected PVC has a lower chlorine concentration at 34% when compared to the rigid PVC, which contains 56 to 58% chlorine [2,23]. The weight-loss profile of untreated PVC also showed that the leftover solid weight after the first pyrolysis stage was 56% and this value was close to the experimental solid yield (57.8%) obtained with 96 s of treatment time; this suggests that this process has completed the first pyrolysis stage (dechlorination) and the second pyrolysis stage has not been achieved.

In addition, the weight-loss profile of de-Cl residue displayed very minimal weight loss (2.5%) between 175 and 350°C (the temperature range for the dechlorination stage) which again indicated that most of the chlorine in the PVC had been removed through the microwave pyrolysis process. These results correlate with the significant decrease of absorbed power and loss tangent when chlorine is continuously removed from the polymer followed by reaching the stabilization stage (where absorbed power and loss tangent remain constant at a very low value) when most of the chlorine had been selectively removed from the polymer.

#### (iv) Chemical bonds and structure via FTIR analysis

**Figure 6.**
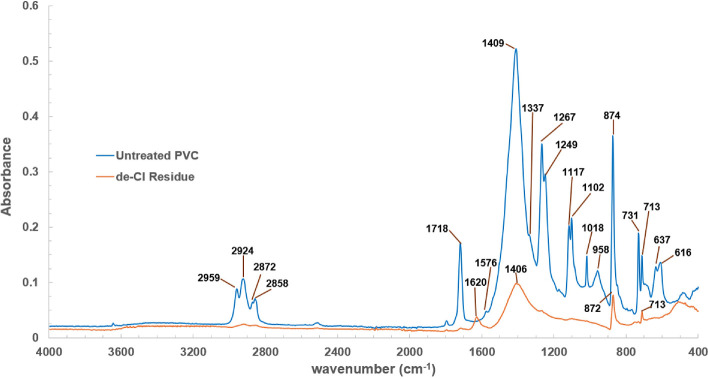
The IR spectra of untreated PVC and de-Cl residue.

The IR spectra of the untreated PVC and de-Cl residue are presented in figure 6 and the assignment of each band is listed in table 2.

**Table 2.** Assignment of the IR bands of untreated PVC and de-Cl residue.

	component	wavenumber (cm^–1^**)**	band assignment
untreated PVC	PVC [13,43,44]	2959	C-H stretching in CHCl
		2924	C-H stretching in CH_2_
		1425	C-H_2_ bending
		1337, 1267, 1249	C-H bending in CHCl
		1102	C-C stretching of PVC backbone
		958	C-H_2_ rocking
		637, 616	C-Cl stretching of secondary chlorides (CHCl) [45]
	phthalate plasticizer [44,46]	2959, 2924, 2872, 2858	C-H stretching
		1718	C=O stretching in O=C-O
		1576	C=C stretching of benzene ring
		1337	C-H bending
		1267	C-O stretching in O=C-O
		1117	C-O stretching
		731	C-H bending of ortho-disubstituted benzene ring
	calcium carbonate [46,47]	1409	anti-symmetry stretching (ν3)
		874	the out-plane band (ν2)
		713	the in-plane band (ν4)
	silicone additive [48]	1018	Si-O-Si band
de-Cl residue	calcium carbonate [46,47]	1406	anti-symmetry stretching (ν3)
		872	the out-plane band (ν2)
		713	the in-plane band (ν4)
	aromatic compound [13,23]	1620	C=C stretching of an aromatic ring
		872	=C–H out-of-plane vibration in aromatic rings

The IR spectrum of untreated PVC contained bands corresponding to pure PVC, and common additives including phthalate plasticizer, calcium carbonate (CaCO_3_) known as calcite and silicone additive. Other minor additives are possibly present in the untreated PVC such as colour pigments and flame retardants [49]. However, these additives are present in small concentrations making them difficult to detect through the FTIR analysis due to weak IR bands and overlapping with other compounds [50]. The structures of PVC, some phthalate plasticizers that are commonly used in flexible PVC such as dinonyl phthalate (DNP), diisononyl phthalate (DINP) and diisooctyl phthalates (DIOP) and calcite are listed in electronic supplementary material, table S1.

It is well-documented that two of the most influential additives in flexible PVC are plasticizers and fillers [4]. Phthalate plasticizer is often added to PVC products to soften PVC and improve PVC flexibility. Although phthalate plasticizer was detected in the IR spectrum of untreated PVC (figure 6), the identification of specific phthalates was difficult due to similarities in the main body structure (as listed in electronic supplementary material, table S1), resulting in very similar IR bands. The most common type of filler is calcite because it is inexpensive, consistent, readily available and more versatile than many other fillers [4]. The addition of calcite can improve the mechanical properties (tensile strength and elongation), thermal stability and electrical properties of PVC products [46]. For the PVC cable compounds (such as the PVC selected for this study), the weight ratio for the composition of calcite to PVC could be 0.5−1:1 [51]. Silicone additive is also used in PVC as a lubricant and for easy demoulding of the polymer [52].

The comparison of the IR spectra between untreated PVC and de-Cl residue in figure 6 clearly shows that all the bands involving chlorine atoms, such as the bands at 616 and 637 cm^−1^ corresponding to the stretching of C–Cl bonds, 1249 to 1337 cm^−1^ attributed by the C-H bending in CHCl, and 2959 cm^−1^ originated by the stretching of the C–H bonds of carbon atoms bonded to a chlorine atom (CHCl) [43,44] were not present in the de-Cl residue as a consequence of the PVC dechlorination achieved by microwave pyrolysis.

Other peaks associated with PVC such as the C-H_2_ rocking at 958 cm^−1^, C-C stretching of PVC backbone at 1102 cm^–1^, and C-H stretching in CH_2_ at 2924 cm^−1^ were not seen in the spectrum of de-Cl residue indicating that PVC was decomposed successfully via microwave pyrolysis process. All the bands linked to the phthalate plasticizers and silicone additive were hardly detected in the spectrum of de-Cl residue; this implies that most of the plasticizers and silicone additives present in the untreated PVC were removed via microwave pyrolysis.

On the other hand, the spectrum of de-Cl residue revealed that the de-Cl residue contains mainly calcite because three bands associated with calcite (713, 872 and 1406 cm^−1^) were observed. This suggested that calcite was not decomposed during the microwave pyrolysis process because it is a stable compound, and its thermal decomposition takes place at high temperatures between 700 and 800°C [53], which is far beyond the microwave pyrolysis process temperature.

A new small band was noticed at 1620 cm^−1^ in the de-Cl residue spectrum, and this is anticipated to be C=C stretching vibration from aromatic compounds [13,23] which are formed from dechlorinated PVC when the C–Cl bonds are broken [21] during the microwave dechlorination process. As C=C bonds linked to aromatic compounds are formed during the pyrolysis process, the band at 872 cm^−1^ in the de-Cl residue may be attributed to aromatic products, specifically to the =C–H out-of-plane vibration in aromatic rings [13,23]. Usually, the dechlorinated PVC is converted to aromatics in the second pyrolysis stage (350 to 525°C). However, it has been reported that the intramolecular cyclization or aromatization reactions may take place during the dechlorination process at approximately 300°C, which leads to the observation of the initial formation of aromatic structure [13,23] in the first pyrolysis (dechlorination) stage. In summary, the IR analysis showed that the de-Cl residue consists mainly of calcite and probably traces of aromatic compounds.

#### (v) Crystalline compound in the de-Cl residue via XRD analysis

The XRD pattern of the de-Cl residue as presented in electronic supplementary material, fig. S4 denotes that the crystalline compound in the de-Cl residue is predominantly calcite (a common filler in PVC [4]) because all the diffraction peaks from the de-Cl residue matched well with the calcite compound in the library database.

#### (vi) Chemical elements in the de-Cl residue via XRF and elemental analysis

The main elements in the untreated PVC and de-Cl residue were determined via XRF and elemental analysis, the results are listed in electronic supplementary material, table S2. The detected elements could be used to indicate the possible compounds in the de-Cl residue as follows: 60.4 wt% calcite (calculated based on 24.2 wt% calcium), 31.9 wt% carbon, 0.3 wt% titanium dioxide (calculated based on 0.2 wt% titanium) and 2.9 wt% hydrogen. The sum of calcite, carbon, titanium dioxide and hydrogen gives a total of 95.5 wt%; the remaining 4.5 wt% is possibly contributed by the undetected elements. The carbon residue in the de-Cl residue is probably amorphous as it is not detected by XRD analysis. Electronic supplementary material, table S2 lists the carbon (36.6 versus 31.9 wt%) and hydrogen (4.4 versus 2.9 wt%) elements in the untreated PVC, showing that they were higher than the de-Cl residue, which is believed to be contributed by the plasticizer in the untreated PVC,while most of the plasticizer in the de-Cl has been removed during the process which is indicated by the IR analysis.

### (c) Preliminary identification of main compounds in liquid products via GC-MS

**Figure 7.**
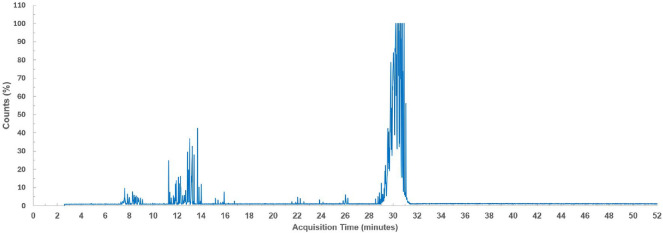
Chromatogram for the liquid product.

Approximately 118 mg of the liquid product collected from the experiment with 96 s of treatment time (6.7% liquid yield in figure 3) was analysed with GC-MS to preliminary identify the main compounds. The chromatogram is shown in figure 7 and the detected compounds are listed in electronic supplementary material, table S3.

The chromatogram, as shown in figure 7 reveals three distinct complex regions of hydrocarbons. The first region is located between 7.3 and 9.1 min, consisting of branched and straight chain C_9_H_18_ alkenes. The second region is located between 11.3 and 13.8 min, containing chain alkenes such as 2,3,3-trimethyl−1-hexene, branched cyclic alkanes such as 2-ethyl−1,1-dimethylcyclopentane, branched and straight chain alcohols such as 6-methyl−1-octanol, and chlorinated species such as 1-chlorononane. All these identified compounds are anticipated as the pyrolytic products formed during the PVC dechlorination and decomposition process in the first pyrolysis stage [54,55].

It is proposed that during the microwave dechlorination process in the first pyrolysis stage, the PVC polymer is decomposed into a range of intermediate products including chain hydrocarbons, chlorinated hydrocarbon fragments, hydrogen and chlorine radicals. Hydrogen radicals may recombine with each other to form hydrogen or react with chlorine radicals to produce HCl, which is the main volatile product in the first pyrolysis stage [1]. The intermediates of alkenes and alkanes may undergo hydrogenation and dehydrogenation reactions to convert to each other, whilst chlorination of alkanes/alkenes may produce chlorinated hydrocarbons [1]. In addition, cyclic hydrocarbons and aromatics are probably formed due to the molecular rearrangements and cyclization/aromatization reactions of unstable polyene fragments formed by dechlorinated PVC [13,22]. Alcohols were detected in the second region and similar results were reported in the literature [56]; however, it is unclear whether the alcohols originate from the additives in PVC or whether they are formed during the pyrolysis process, and this requires further investigation.

The third region is located between 28.5 and 31.1 min, comprising a mix of phthalates. The identified phthalates included DNP, DINP and DIOP. These phthalates are commonly used as a plasticizer in PVC in the production of hoses, wires and cables to improve flexibility and UV resistance [57–59]. Hexadecanoic acid—pentyl ester (C_21_H_42_O_2_) was identified in the third region, and this compound is probably grafted on to the PVC chain as the internal plasticizer [59].

In summary, the IR and GC-MS analysis results showed that most phthalates are probably removed through the microwave dechlorination process and then recovered in the liquid product. It is reported in the literature that phthalate plasticizers are easily thermally desorbed during the pyrolysis process because they are not chemically bound to the PVC structure [60].

## 4. Implications from the key findings and the future outlook

The results presented in this study demonstrate that the microwave pyrolysis process has removed chlorine and most plasticizers from the PVC efficiently. This suggests that microwave pyrolysis is a promising technology to decompose and dechlorinate flexible PVC. The feasibility of this technology to dechlorinate rigid PVC and the mixture of different types of PVC will be investigated in the near future.

Even though the loss tangent of PVC is low at room temperature and below its T_g_ (55°C), its loss tangent increases rapidly with rising temperature after crossing the T_g_ due to the polar chlorine group within the PVC structure and possibly due to the presence of polar phthalate plasticizers in the PVC that improves the chain mobility. It has been reported that the amount of dissipated power during microwave heating is directly proportional to the loss factor [36], therefore the significant increase of loss tangent from T_g_ to the peak temperature at 260°C for the untreated PVC (0.008 to 0.1 in figure 4) potentially leads to a corresponding enhancement in the heating rate, which enables the PVC to be heated rapidly and directly within the entire polymer volume in a short treatment time.

On the other hand, dechlorination of PVC through other heating processes such as conventional pyrolysis is time-consuming and energy-intensive because the heat is transferred inwards from a heating source such as a furnace (high temperature) to the reactor wall, and then to the polymer (low temperature) in the presence of a thermal gradient via conduction and convection heat transfer [33]. This is predicted as one of the reasons restricting the commercialization of conventional pyrolysis for PVC dechlorination.

Furthermore, this new process is simple and environmentally friendly, without the requirement of either a solvent or a microwave absorber, providing significant advantages over other reported dechlorination methods listed in table 1. A direct comparison of this work with the literature results listed in table 1 cannot be made due to different PVC samples and different dechlorination methods; however, general observations can be made. The dechlorination efficiency achieved by the microwave pyrolysis process in this study was higher than the dechlorination efficiencies achieved by different dechlorination methods listed in table 1 (99.6% versus 90 to 98.2%). In addition, it is expected that this process will have a lower capital burden than other dechlorination methods owing to the significantly shorter treatment time (96 s versus half an hour to a few hours) that may reduce energy requirements due to less heat loss to the surrounding and no requirement of a microwave absorber/solvent, which eliminates the costs associated with downstream absorber/solvent separation process. Even though this process is proven effective at the laboratory scale, it is essential to scale the process to a larger size to validate its efficiency to process large throughput and conduct techno-economic assessment and life-cycle analysis to evaluate its economic viability and carbon footprint, respectively.

A sustainable and economically feasible route for PVC recycling is proposed here by combining PVC decomposition and dechlorination via microwave pyrolysis with the generation of positive values from the pyrolysis products; the proposed route is illustrated in electronic supplementary material, fig. S5. More details about the potential values of the pyrolysis products are provided below.

— Generation of valuable products from de-Cl residue: Calcite is found to be the main component in de-Cl residue and calcite is widely added into asphalt binder for the improvement of mechanical and physical properties [61,62], therefore the de-Cl residue has high potential to be used as a modifier/binder in the production of high-value road surfacing asphalt mixtures. This is envisaged to create a positive effect by mitigating some of the carbon emissions associated with asphalt production, which aligns and supports the achievement of net-zero (decarbonization) road material targets in the UK by 2040 [63].
— Plasticizer recycling: Phthalate plasticizers have been regarded as hazardous compounds in numerous reports based on their toxicological effects, including bioaccumulation potential, endocrine disruption, carcinogenicity and developmental defects [64]. Furthermore, phthalates are not chemically bound to products and thus they are easily released into the environment, resulting in human exposure. A sustainable plasticizer recycling route is highly important; however, no studies have been reported thus far. Therefore, the potentiality to extract plasticizers from the liquid product for reuse in PVC production is important and should be investigated.
— Chlorine recovery: The HCl captured by the water could be recovered and potentially used in industrial applications such as for pickling of steel to remove the impurities and rust (iron oxide) from alloy, iron and steel surfaces to prepare the steel for the final applications in building and construction projects [65]. The recovered HCl could be used for oil well acidizing whereby the acid is injected into rock formation of oil wells to dissolve rock, which creates a larger porous body to help extract existing oil [65].

## 5. Conclusions

We present a novel microwave pyrolysis process that could decompose and dechlorinate problematic PVC rapidly and effectively without requiring a solvent/microwave absorber. PVC is readily heated under microwaves due to the polar chlorine groups within its structure and the presence of polar phthalates. The dielectric analysis shows that the de-Cl residue had close-to-zero loss tangent across temperatures from 20 to 400°C, the IR analysis shows that all the bands associated with C-Cl stretching were not detected in the de-Cl residue, and the thermogravimetric analysis reveals that de-Cl residue had minimal weight loss (2.5%) within the dechlorination temperature range (175 to 350°C); all demonstrating the achievement of dechlorination through microwave pyrolysis.

This process shows notable advantages over other literature-reported dechlorination methods such as solvent dehalogenation, hydrothermal treatment and conventional pyrolysis in terms of high dechlorination efficiency (greater than 99% chlorine removal efficiency), short treatment time in 96 s, simple operation under atmospheric pressure without the requirement of any additives to the process. This demonstrates the potential of the microwave pyrolysis process to make recycling of problematic PVC economically feasible. Furthermore, the pyrolysis products and de-Cl residue have the potential to be converted into valuable products.

## Data Availability

Further data are available on request from the authors. Supplementary material is available online [66].
